# Plasma antioxidant potential measured by total radical trapping antioxidant parameter in a cohort of multiple sclerosis patients

**DOI:** 10.1002/brb3.3377

**Published:** 2024-01-17

**Authors:** Marge Kartau, Joonas Kartau, Marjatta Pohja, Auli Verkkoniemi‐Ahola

**Affiliations:** ^1^ Clinical Neurosciences, Neurology Helsinki University Hospital and Helsinki University Helsinki Finland; ^2^ Department of Mathematics and Statistics University of Helsinki Helsinki Finland; ^3^ Clinical Neurosciences, Neurology Helsinki University Hospital Helsinki Finland

**Keywords:** biomarker, multiple sclerosis, oxidative stress, total radical trapping parameter assay

## Abstract

**Background:**

Oxidative injury has been implicated as a mediator of demyelination, axonal damage, and neurodegeneration in multiple sclerosis (MS). There is a high demand for oxidative injury biomarkers. The aim of the study was to evaluate MS patients’ plasma antioxidant potential using the total radical trapping parameter (TRAP) assay and examine its usefulness as an MS disease biomarker.

**Methods:**

A total number of 112 MS patients underwent an analysis of TRAP. In addition, plasma uric acid (UA) levels were analyzed. The neurological and radiological data were collected from patient records from Helsinki University Hospital during 2012–2013 when first‐line injectables of moderate‐efficacy, natalizumab (NTZ), and fingolimod (FTY) of high efficacy disease modifying therapies and in some cases azathioprine (AZT) were used to treat MS.

**Results:**

TRAP values were negatively associated with expanded disability status scale (EDSS) score with *p*‐value .052, *β* = −28. There was also a negative association in TRAP values between patients with no medication (*n* = 22, TRAP mean 1255 μmol/L (95% confidence interval [CI] 1136–1374)) and patients who received NTZ, *p*‐value .020 (*n* = 19, TRAP mean was 991 μmol/L (95% CI 849–1133) or FTY treatment, *p*‐value .030 (*n* = 5, TRAP mean 982 μmol/L (95% CI 55–1909). Due to a small sample size, these results were not significant after applying a false discovery rate correction at a 0.05 significance level but are worth highlighting. Men in the study had higher TRAP values, *p*‐value = .001 (TRAP mean 1320 ± 293 μmol/L) than women (TRAP mean 1082 ± 288 μmol/L). UA was positively associated with TRAP values, *p*‐value <.001 and UA levels in men (UA mean 334.5 ± 62.6 μmol/L) were higher compared to women (UA mean 240 ± 55.8 μmol/L), *t*‐test *p*‐value <.001. The significant difference in TRAP levels between genders, with men showing higher TRAP values than women, may be attributed to the variation in UA levels.

**Conclusion:**

Our findings suggest that lower plasma antioxidant potential is linked to more severe disability measured by EDSS scores. Patients treated with NTZ and FTY had reduced antioxidant power, which might be influenced by the active MS disease rather than the treatments themselves. The study reveals a strong positive correlation between UA levels and TRAP, particularly among women. However, men on average had better antioxidant potential than women. Neither the disease type nor the duration influences TRAP levels. While serving as a marker of antioxidant potential, plasma TRAP in MS patients does not reliably reflect overall oxidative stress (OS) and should not be solely used as an indicator of OS.

## Graphical abstract

This study evaluates multiple sclerosis (MS) patients’ plasma antioxidant potential using the total radical trapping parameter (TRAP) assay. Our findings suggest that lower plasma antioxidant potential is associated with higher disability and active MS immunopathology. MS patients’ plasma TRAP does not reflect the total antioxidant defense of the body, and when used as a single plasma biomarker, TRAP is not a reliable marker of oxidative stress in MS disease.

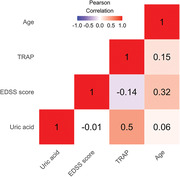

## 1 INTRODUCTION

Multiple sclerosis (MS) is a chronic demyelinating disease of the central nervous system (CNS) in which both inflammatory and neurodegenerative processes occur simultaneously (Wang et al., 2015). The histopathology of the disease is well established, including demyelination, oligodendrocyte loss, activation of microglia and astrocytes, and neuro‐axonal damage. The pathogenesis of MS is complex and still partly uncertain. Oxidative injury has been associated to MS pathogenesis, especially to MS‐related neurodegeneration and smoldering neuroinflammation (Butterfield et al., 2012; de Barcelos et al., 2019; Melo et al., 2011; Moezzi et al., 2022; van Horssen et al., 2010). Oxidative molecules and antioxidant enzymes are found within MS lesions in the CNS (Moezzi et al., 2022; van Horssen et al., 2010). There is accumulating data about oxidative stress (OS) and mitochondrial injury playing a role in the neurodegenerative component of MS (de Barcelos et al., 2019).

Oxidative injury is not specific for MS as it has a major role in numerous neurodegenerative diseases (Butterfield et al., 2012; Melo et al., 2011), but, in MS, it is driven by inflammatory processes and amplified by changes in the brain due to aging and accumulation of disease burden (Lassmann & van Horssen, 2016). Free radicals are overproduced during the inflammatory activity, but also activated microglial cells and CNS infiltrating macrophages produce enzymes that are involved in the production of oxidative molecules (Friese et al., 2014), leading to cells energy failure, apoptosis, and neurodegeneration (Trapp & Stys, 2009; Valko et al., 2007). Antioxidants terminate the oxidation process of free radicals and thus play a key role in preventing oxidative injury and reducing OS (Cadet & Davies, 2017).

The accurate assessment of MS patient oxidative status is a problem due to the lack of adequate biomarkers. With respect to MS patients, many studies have analyzed distinct OS parameters, which are mostly markers of protein oxidation and lipid peroxidation in serum/plasma, CSF, different blood cells, urine, and saliva samples (Acar et al., 2012; Bamm et al., 2015; Fiorini et al., 2013; Guan et al., 2015; Hunter et al., 1985; Karlík et al., 2015; Miller et al., 2013; Naidoo & Knapp, 1992). There are several methods to measure antioxidant capacity. These methods are based on chemical reactions and rely on spectrophotometry, presupposing the occurrence of distinct colors of the analyzed solutions (Munteanu & Apetrei, 2021). Total radical trapping parameter (TRAP) is one of the most employed methods for estimating antioxidant capacity of substances in vitro, and it has been used to examine the antioxidant property of human plasma and other body fluids (Munteanu & Apetrei, 2021; Uotila et al., 1994). TRAP measures the protection provided by antioxidants on the fluorescence decay of R‐phycoerythrin during a peroxidation reaction. TRAP gives a more reliable estimation of serum or plasma antioxidant capacity than the measurement of each known individual antioxidants because it measures the synergistic effect among various antioxidant compounds (Dresch et al., 2009).

TRAP has been used in the evaluation of oxidative status of patients with systemic lupus erythematosus, cancer, hepatitis, preeclampsia, and diabetes (Ceriello et al., 1997; La Vecchia et al., 2013; Scavuzzi et al., 2018; Uotila et al., 1994; Venturini et al., 2010). In MS disease, TRAP has been studied with association to D‐vitamin levels and as a potentially predictive biomarker for disease progression and higher disability (Kallaur et al., 2017; Oliveira et al., 2012, 2017). However, the assessment of patients antioxidant capacity alone is not sufficient to make decisions about OS without additional information on oxidative injury or tissue free radical production (Costantini & Verhulst, 2009).

The aim of this study was to investigate the usefulness of TRAP method as a biomarker in MS disease and neurodegeneration and to study its association with disease modifying therapies (DMTs). Uric acid (UA) is a well‐known water‐soluble antioxidant that contributes significantly to the plasma antioxidant capacity (Nälsén et al., 2006). We measured plasma UA level to determine its contribution to the plasma antioxidative activity.

## 2 METHODS

### 2.1 Study participants and measures

In this retrospective study, clinical and demographic data were collected, and plasma samples were obtained from 112 MS patients. The subjects were recruited during 2012–2013. The data were obtained from Helsinki University Hospital (HUS)’s clinical records. All patients were examined by a neurologist, and they were diagnosed with clinically definite MS according to the 2010 revised McDonald criteria (Polman et al., 2011) and the date of diagnosis and first symptoms were recorded. According to diagnostic criteria, in addition to the clinical picture, diagnose of MS had to be supported by characteristic magnetic resonance imaging (MRI) features and CSF findings. All study patients had a 1.5 T MRI scan and lumbar puncture performed prior to the study.

Diagnoses of MS in the database were classified into three categories: relapsing‐remitting MS (RRMS), secondary progressive MS (SPMS), and primary progressive MS (PPMS). Neurologic disability was assessed with expanded disability status scale (EDSS) (Kurtzke, 1983).

The demographic and clinical information of the participants are presented in Table 1. Patients were divided into 2 groups according to their age: aged 18–40 and 41–72 years.

**TABLE 1:** The demographic and clinical information of participants.

	Number and % of patients		
Characteristic	Men and women	Male	Female
Total	112 (100.0%)	26 (23.0%)	86 (77.0%)
RRMS	94 (83.9%)	20 (76.9%)	74 (86.0%)
SPMS	16 (14.3%)	6 (23.1%)	10 (11.6%)
PPMS	2 (1.8%)	0 (0%)	2 (2.3%)
Age, mean ± sd (range)	41.9 ± 10.35 (18–72)	43.0 ± 11.45 (18–62)	41.5 ± 10.05 (19–72)
EDSS, mean ± sd (range)	3.21 ± 1.84 (0–7)	3.15 ± 1.52 (1–6.5)	3.22 ± 1.93 (0–7)
Smokers/nonsmokers (not all data available)	23/54	4/12	19/42
Duration of disease (range)	0–30^a^	–	–
**Brain imaging**			
MRI taken	112 (100.0%)	26 (100.0%)	86 (100.0%)
Last MRI taken within 2 years (*n*, %)	72 (63.2%)	–	–
OCBs positive at the time of diagnosis (*n*, %)	47 (96.1%)	–	–
CSF IgG‐index over the normal at the time of diagnosis (*n*, %)	56 (88.8%)	–	–
**Use of DMT**			
Without DMT	22 (19.6%)	6 (23.1%)	16 (18.6%)
Total receiving any DMT	90 (80.4%)	20 (76.9%)	70 (81.4%)
Beta‐interferons	42 (37.5%)	10 (38.5%)	32 (37.2%)
Glatiramer acetate	19 (17.0%)	7 (26.9%)	12 (14.0%)
Fingolimod	5 (4.5%)	0 (0%)	5 (5.8%)
Natalizumab	19 (17.0%)	3 (11.5%)	16 (18.6%)
Azathioprine	4 (3.6%)	0 (0%)	4 (4.7%)
Mitoxantrone	1 (0.9%)	0 (0%)	1 (1.2%)

*Note*: Some data were unavailable from certain patients.

Abbreviations: DMT, disease modifying therapy; EDSS, expanded disability status scale; MRI, magnetic resonance imaging; MS, multiple sclerosis; PPMS, primary progressive MS; RRMS, relapsing‐remitting MS; SPMS, secondary progressive MS.

^a^ Five patients were newly diagnosed with MS.

Vitamins intake was assessed by a self‐reported questionnaire about daily vitamins supplementation.

The study was approved by the Ethics Committee of the HUS and conducted according to the Declaration of Helsinki. Informed written consent was received from each study participant.

### 2.2 Analytical methods

TRAP tests were performed in the UTULab in Turku. TRAP was performed as previously published (Lissi et al., 1995). TRAP values of samples were obtained using an equation of standard curve of Trolox dilutions. Trolox, a vitamin E analog, served as a standard radical scavenger. TRAP measures the chain‐breaking potential to reduce peroxyl radicals generated by AAPH/ABAP (2,2′‐azobis(2‐amidinopropane) dihydrochloride) and represents a measurement of the hydrogen atom transfer mechanism (Polman et al., 2011). There are no population studies conducted with large patient cohorts for TRAP, but there are smaller cross‐sectional and intervention studies available. The reference values for healthy persons plasma TRAP levels range from 610 to 1620 μmol/L (Ceriello et al., 1997; La Vecchia et al., 2013; Scavuzzi et al., 2018; Venturini et al., 2010).

Plasma UA was measured with an enzymatic assay on a spectrophotometer at the HUSLab.

### 2.3 Statistical analysis

Statistical analyses were performed in R version 4.2.2.

The variables investigated for this study were as follows: EDSS score, MS type, relapses, DMTs, alcohol use, vitamins intake, and smoking habits. We combined IFNb‐ and glatiramer‐treated patient samples because these two treatments are both used as first‐line therapies for MS and are categorized as immunomodulators.

**FIGURE 1:**
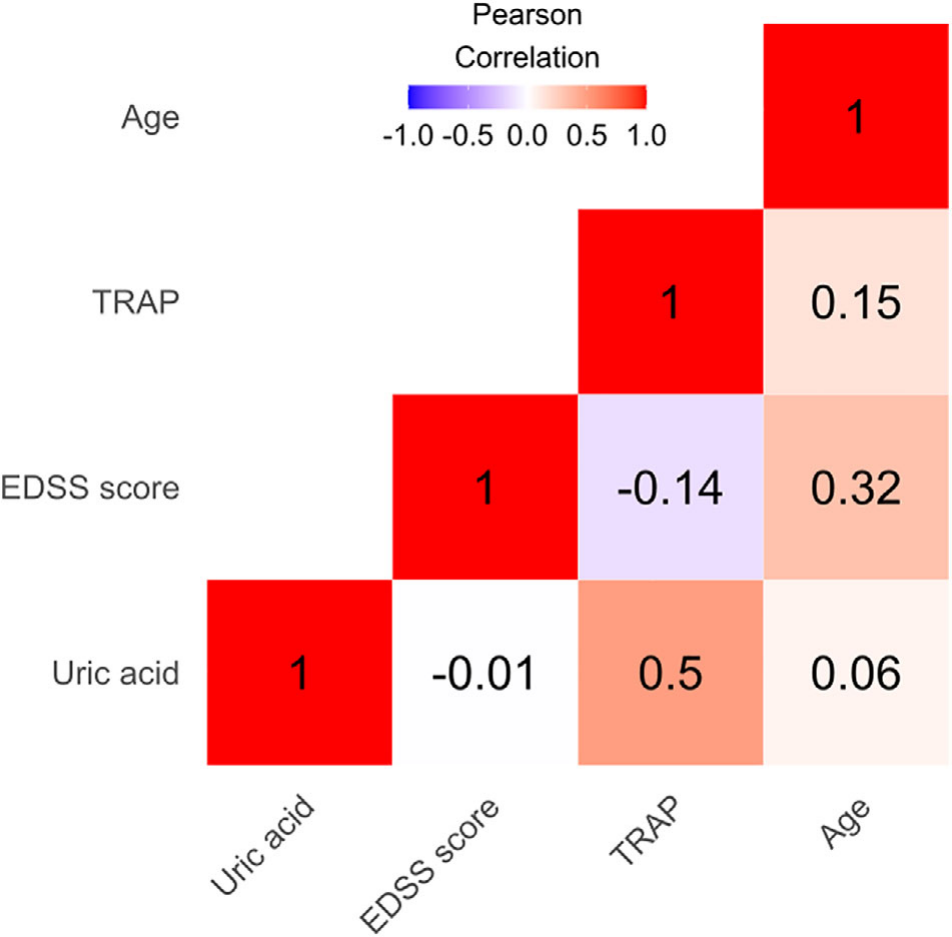
Correlation heatmap for the continuous variables: uric acid (UA), expanded disability status scale (EDSS) score, total radical trapping parameter (TRAP), and age. The intensity of color indicates the strength and direction of correlation: Red colors represent positive correlations; blues represent negative correlation.

A correlation heatmap was created to visualize the relationship between continuous measurements such as UA, EDSS score, TRAP, and age (Figure 1).

*T*‐tests were performed to assess differences between UA and TRAP levels between men and women.

To assess the associations between measurements of interest and TRAP, we use linear regression and ANCOVA models. Starting with a base model regressing TRAP against sex, age, and log(UA), we added each measurement of interest one at a time to the model (EDSS score, MS type, relapses, DMT, alcohol use, and smoking) and recorded the associated *p*‐values, effect sizes, and their 95% confidence intervals (CI).

For UA, the log transformation was performed because its distribution was right skewed. As the effect of UA on TRAP levels has been noted in previous studies, we added it to the baseline model to adjust for confounding effects (Fabbrini et al., 2014; Nguyen‐Khoa et al., 1999).

The Benjamini–Hochberg method was used to adjust the *p*‐values at a .05 significance level.

## 3 RESULTS

### 3.1 Descriptive statistics

The study had a total of 112 patients, and most patients were women (women *n* = 86, 77.0%; men *n* = 26, 23.0%). There were 94 (83.9%) RRMS, 16 (14.3%) SPMS, and 2 (1.8%) PPMS patients.

During the previous 2 years, 39 (34.8%) of patients have had at least 1 MS relapse, and 72 (64.3%) patients had no relapse. Six (5.1%) patients had recent relapse within 2 months. No data about relapse rate were available for one patient.

During our study, injectable interferons (*n* = 42, 37.2%) and glatiramer acetate (*n* = 19, 17.1%) were available DMTs for MS as a first‐line treatment, whereas NTZ (*n* = 19, 17.1%) and FTY (*n* = 5, 4.5%) were available for second‐line treatment. One patient (0.9%) had mitoxantrone treatment. Four (3.6%) were using AZA.

### 3.2 TRAP

The overall TRAP range for all patients was 241–1735 μmol/L. The mean value of TRAP was 1137 μmol/L (range 241–1735 μmol/L, median 1156 μmol/L), 95% CI 1080.11–1194.21 (Table 2). Six patients had TRAP values lower than 610 μmol/L, and six had higher than 1620 μmol/L.

**TABLE 2:** Trap mean values.

	Mean TRAP (μmol/L) ± sd (range)		
	All	Men	Women
Overall	1137 ± 305 (241–1735)	1320 ± 293 (241–1735)	1082 ± 288 (266–1713)
**Age (years)**			
18–40	1121 ± 337 (241–1735)	1301 ± 430 (241–1735)	1072 ± 295 (409–11637)
41–72	1149 ± 281 (266–1713)	1331 ± 179 (1017–1656)	1089 ± 284 (266–1713)
**Type of MS**			
RRMS	1120 ± 311 (241–1735)	1307 ± 329 (241–1735)	1070 ± 288 (266–1681)
SPMS	1229 ± 265 (761–1713)	1361 ± 131 (1183–1558)	1151 ± 299 (761–1713)
PPMS	1183 ± 297 (973–1393)	–	1183 ± 297 (973–1393)
**Relapses in a 2‐year period**			
Relapse	1113 ± 258 (543–1634)	1324 ± 199 (1017–1634)	1047 ± 240 (543–1459)
No relapse	1151 ± 329 (241–1735)	1318 ± 338 (241–1735)	1100 ± 312 (266–1713)
**EDSS score**			
EDSS ≥ 3	1127 ± 320 (241–1713)	1290 ± 330 (241–1656)	1065 ± 296 (293–1713)
EDSS < 3	1150 ± 288 (266–1735)	1375 ± 213 (1084–1735)	1101 ± 280 (266–1681)
**Smoking**			
Smokers	1189 ± 274 (543–1735)	1333 ± 312 (1084–1735)	1158 ± 265 (543–1637)
Non smokers	1160 ± 294 (266–1713)	1430 ± 123 (1209–1659)	1083 ± 283 (266–1713)
**MRI lesions in a 2‐year period**			
No new lesions	1121 ± 289 (293–1656)	1316 ± 212 (1074–1656)	1054 ± 285 (293–1505)
New T2 and Gd lesions	1110 ± 295 (241–1459)	901 ± 610 (241–1444)	1155 ± 195 (721–1459)
New T2 lesions	1096 ± 303 (241–1634)	1234 ± 480 (241–1634)	1054 ± 229 (543–1459)
**DMT**			
No medication	1255 ± 268 (761–1713)	1426 ± 152 (1209–1634)	1190 ± 276 (761–1713)
Interferon and glatiramer acetate	1159 ± 294 (241–1735)	1279 ± 334 (241–1735)	1113 ± 267 (266–1681)
Natalizumab	991 ± 294 (543–1539)	1337 ± 280 (1017–1539)	926 ± 255 (543–1325)
Fingolimod	982 ± 473 (293–1459)	–	982 ± 473 (293–1459)
Azathioprine	968 ± 191 (718–1153)	–	968 ± 191 (718–1153)

Abbreviations: DMT, disease modifying therapy; EDSS, expanded disability status scale; MS, multiple sclerosis; MRI, magnetic resonance imaging; PPMS, primary progressive MS; RRMS, relapsing‐remitting MS; SPMS, secondary progressive MS.

The correlation between UA levels and TRAP values is strongly positive (*ρ* = .500) across the entire study cohort (Figure 1). However, this correlation's strength is influenced by gender. In the case of female participants, there is a strong positive correlation (*ρ* = .496) between UA and TRAP values. This indicates that in women, higher UA levels are linked to higher TRAP values, which suggests a collaborative effect on antioxidant protection. On the other hand, among male participants, the correlation between UA and TRAP values is less pronounced (*ρ* = .132). This suggests that although there might not be a strong linear relationship between UA and TRAP in men, other factors related to gender might be influencing their higher TRAP values.

**FIGURE 2:**
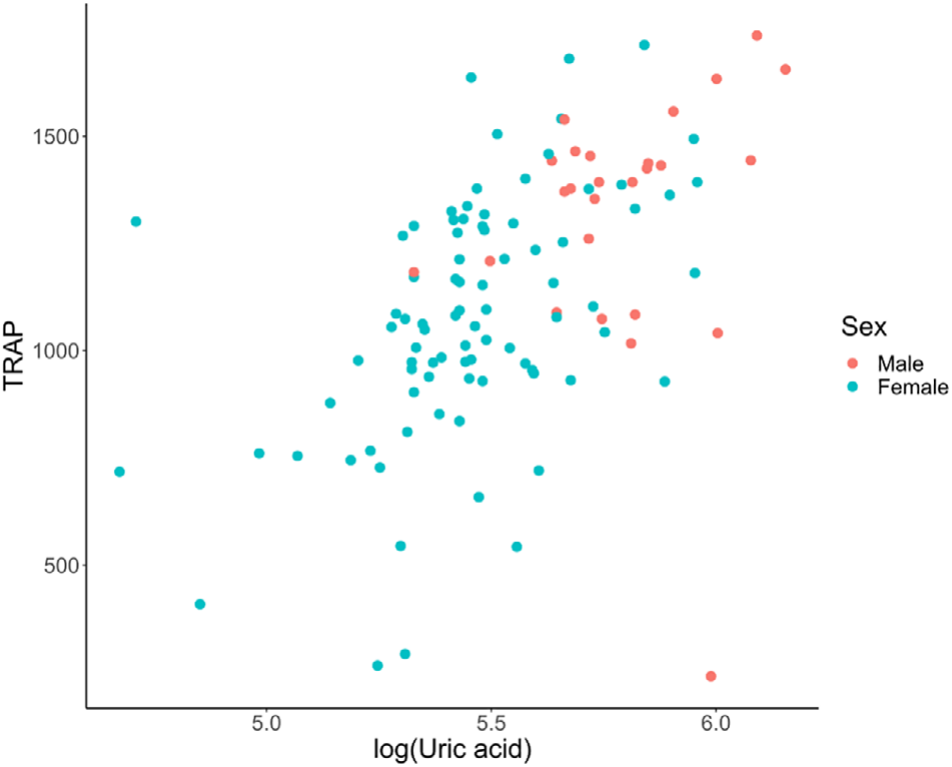
Scatter plot of total radical trapping parameter (TRAP) plotted against log(UA).

When comparing the TRAP values of men (TRAP mean 1320 ± 293 μmol/L) and women (TRAP mean 1082 ± 288 μmol/L), it becomes clear that men have significantly higher TRAP values, with a *p*‐value of .001. This difference could be attributed to the fact that men also have higher UA levels compared to women, as indicated by the mean UA values (334.5 μmol/L for men vs. 240 μmol/L for women), and this difference is statistically significant with a *p*‐value of less than .001, as shown in Figure 2. The relationship between UA and sex is highlighted in Tables 3 and 4. In in the former, we list the effects sizes and *p*‐values of the model that does not include log(UA), and in the latter, we include log(UA) to assess its influence on the observed associations. Considering the complexities involved, it is plausible that the higher TRAP values observed in men may be influenced by a combination of factors beyond the direct linear correlation between UA and TRAP levels.

**TABLE 3:** Effect sizes and *p*‐values for the covariates in the model, including age and sex as covariates and total radical trapping parameter (TRAP) levels as the response.

Variable	Effect size (95% CI)	*p*‐Value
Age	3.75 (−1.48, 8.98)	.158
Sex (male as baseline)	−236 (−364, −108)	<.001

**TABLE 4:** Effect sizes and *p*‐values for the covariates in the baseline model with total radical trapping parameter (TRAP) levels as the response.

Variable	Effect size (95% CI)	*p*‐Value
Age	3.256 (−1.53, 8.05)	.181
Sex (male as baseline)	−60.469 (−199.05, 78.11)	.389
Log(UA)	517.009 (297.99, 736.03)	<.001

Abbreviation: UA, uric acid.

Upon controlling for age, a noteworthy pattern emerges: Initially, a significant association between TRAP and sex is observed (*p*‐value < .001); however, with the inclusion of UA in the model, the *p*‐value increases to .389, whereas UA is instead significantly associated (*p*‐value < .001), indicating a confounding effect of UA on the relationship.

Disease type (RRMS, SPMS, or PPMS), relapse rate, duration of disease, and smoking did not significantly affect TRAP values (Figure 1). A summary of the TRAP mean values can be seen in Table 2.

The effects of EDSS and age on TRAP were potentially masked in the data because they are positively correlated with each other (*ρ* = .32, Figure 1) but have opposite signed effects. Age on average was higher in subjects with high EDSS compared to patients with a low EDSS score but increases in EDSS score decreased TRAP values (*β* = −28.211, *p*‐value = .052), whereas higher age increased TRAP values (*β* = 4.866, *p*‐value = .056), see Table 5.

**TABLE 5:** Effect sizes and *p*‐values for the covariates in the baseline model, including expanded disability status scale (EDSS) score with total radical trapping parameter (TRAP) levels as the response.

Variable	Effect size (95% CI)	*p*‐Value
Age	4.866 (−0.13, 9.86)	.056
Sex (male as baseline)	−61.971 (−198.74, 74.81)	.371
EDSS	−28.21082 (−56.61, 0.190)	.052

Abbreviation: EDSS, expanded disability status scale.

There were notable differences in TRAP values between patients receiving specific DMTs and those without medication. Specifically, a significant difference in TRAP values was identified with a *p*‐value of .02 between patients without medication (TRAP mean 1255 μmol/L) and patients on NTZ medication (TRAP mean 991 ± 294 μmol/L, 95% CI 849–1133). Furthermore, a similar observation was made between patients without medication and those on FTY medication, with a *p*‐value of .03 (TRAP mean 982 μmol/L, 95% CI 55–1909) (Table 6).

**TABLE 6:** Effect size and *p*‐values for the discrete covariates added one at a time to the baseline model with total radical trapping parameter (TRAP) levels as the response.

Variable	Baseline/comparison	Change in mean from baseline (95% CI)	*p*‐Value
MS type	RRMS/SPMS	−27.4 (−194, 140)	.746
	RRMS/PPMS	−79.6 (−480, 321)	.695
Relapses	No/yes	86.7 (−135, 309)	.440
DMT	No DMT/natalizumab	−200 (−366, −34.4)	.018
	No DMT/azathioprine	−185 (−467, 96.5)	.195
	No DMT/fingolimod	−294 (−550, −37.3)	.025
	No DMT/interferons	−102 (−229, 24.9)	.114
Alcohol	Moderate use/heavy use	−44.8 (−154, 65.0)	.419
Smoking	No/yes	15.6 (−103, 134)	.795

*Note*: Benjamini–Hochberg method was used to adjust the *p*‐values at a .05 significance level.

Abbreviations: DMT, disease modifying therapy; PPMS, primary progressive MS; RRMS, relapsing‐remitting MS; SPMS, secondary progressive MS.

It is important to emphasize, however, that after a correction for multiple comparisons using the false discovery rate at a significance level of 0.05, these observed differences in TRAP values for NTZ and FTY medication did not retain statistical significance. Therefore, although initial differences were detected, these findings suggest that the variations in TRAP values for NTZ and FTY medication may have arisen due to other factors. Furthermore, it is worth noting that no statistically distinguishable differences in TRAP values were found for all other DMTs investigated in this study.

## 4 DISCUSSION

In our study, we found that the antioxidant capacity of MS patients’ plasma was predominantly influenced by their sex, and, to some extent, by the efficacy of the DMTs, they were receiving, as well as their disability level. Our data revealed a decrease in TRAP levels among patients undergoing NTZ and FTY treatments, which are used for treatment of highly active MS disease. This suggests that antioxidant capacity might decrease in these treatment groups. NTZ, a treatment for RRMS, has various effects beyond just reducing inflammation. It can also affect OS, catecholamine levels, and melatonin levels, as shown in studies by Tasset et al. and Escribano et al. (Bahamonde et al., 2014; Escribano et al., 2016; Tasset et al., 2012, 2013). The decrease in TRAP levels might arise from more severe forms of MS or chronic inflammation that is accompanied by increased OS. However, a decrease in plasma antioxidant capacity may not necessarily be unfavorable if it signifies decreased free radical production and a reduced need for antioxidants. To gain better insights into the link between oxidative status and DMTs, repeated TRAP measurements before and after treatment initiation could provide valuable information.

We observed comparable TRAP levels between patients not taking any medication and those treated with moderate‐efficacy DMTs such as interferons or glatiramer acetate. Although this suggests a potentially better oxidative potential among patients on these treatments, reflecting less aggressive forms of MS, it is essential to acknowledge that variations in TRAP levels might also be influenced by the specific DMTs utilized. This suggests that the type of medication prescribed could potentially lead to higher or lower TRAP values, indicating varying degrees of antioxidant capacity. Consequently, high or low TRAP values may arise from different prescribed medications. We did not have the opportunity to measure plasma oxidative potential before and after patients started their DMTs, and future studies should aim to collect this data if possible. Another issue was the high heterogeneity among the study participants limiting comparability across our study.

The significant difference in TRAP levels between genders, with men showing higher TRAP values than women, may be attributed to the variation in UA levels (Halperin Kuhns & Woodward, 2020). UA, which contributes substantially to the scavenging of free radicals in plasma, is strongly correlated to TRAP (Oliveira et al., 2012). Our study reveals that the positive correlation between UA and TRAP was specifically evident in the women's group. The association between sex and plasma antioxidant potential is interesting finding. This adds a new angle to how diseases related to OS can affect men and women differently (Kander et al., 2017). When it comes to MS, gender‐related differences are widely recognized; men exhibit lower susceptibility and a distinct disease course compared to women, with a female‐to‐male ratio of approximately 3:1 (Boström et al., 2013; Orton et al., 2006; Voskuhl, 2018). There are also qualitative differences in the disease development between sexes, with male patients experiencing increased disease progression, brain atrophy, and cognitive impairment (Savettieri et al., 2004; Voskuhl et al., 2020; Weinshenker et al., 1991). There is evidence that estrogen, progesterone, and testosterone hormones have function in immunomodulation and neuroprotection in the CNS (Airas, 2015), and data of previous studies suggest that sex hormone‐dependent differences in OS may play an important role in many vascular and neurodegenerative diseases (Frey & Dias, 2014; Miller et al., 2007). Further investigation is needed to study underlying mechanisms driving this gender‐based disparity in TRAP values.

Smoking did not significantly affect TRAP values in MS patients. This may be explained by the fact that most smokers in this study had considerably reduced cigarette smoking after the MS diagnose.

To advance future research, an improved understanding of the changes in oxidative status and antioxidant capacity induced by DMTs could be achieved through the analysis of larger patient cohorts and taking samples before and after treatment. It would be interesting to study the influence of newer DMTs (monoclonal antibodies, new oral DMTs) to patient oxidative status. In addition to plasma or serum samples, the CSF TRAP may add to the understanding of antioxidative response in MS disease.

Plasma concentrations of certain antioxidants may influence TRAP values. Vitamins C, E (α‐tocopherol), and UA are known to significantly contribute to TRAP (Kanter et al., 1993; Kaur & Halliwell, 1990; Vasankari et al., 1997). Vitamins D and C were the most commonly used vitamins. Some patients used multivitamins containing very small doses of vitamin E. The investigation into vitamins C and E was inconclusive.

Neurodegeneration in MS progresses stealthily over a long period, but the treatment window is short, mainly the prodromal stage before evident cognitive and motor symptoms. In younger patients, neurodegeneration may be clinically silent because of brain reserve capacity and plasticity (Sandi et al., 2021). However, the compensation finally fails, and the disease process eventually leads to permanent disability. Therefore, biomarkers which could also recognize underlying smoldering pathologies are needed to measure the total disease activity. Biomarkers could also help to stratify different disease phenotypes and ensure that new drugs are reaching the appropriate target. Better understanding of OS contribution in MS pathology in different disease stages, and types will promote the development of novel targeted therapies for remyelination and enable the implementation of neuroprotective therapies. Despite the limitation of using TRAP to measure the total antioxidant defense of the body, it may be an informative marker before and during DMT medications in combination with other biomarkers. Antioxidant capacity parameter could be useful as an initial test for the diagnosis of OS and monitoring therapy response, but a single measurement of antioxidant status is not sufficient.

## AUTHOR CONTRIBUTIONS

Auli Verkkoniemi‐Ahola and Marge Kartau devised the projectand the main conceptual ideas. Joonas Kartau together with Marge Kartau did the numerical calculations and statistical work for the study. Marge Kartau, Marjatta Pohja and Auli Verkkoniemi‐Ahola did the clinical study. Marge Kartau wrote the manuscript. All authors discussed the results and contributed to the final manuscript.

## CONFLICT OF INTEREST STATEMENT

The authors declare no conflicts of interest.

## FUNDING INFORMATION

No external funding was available for this study.

### PEER REVIEW

The peer review history for this article is available at https://publons.com/publon/10.1002/brb3.3377.

## Data Availability

The data that support the findings of this study are available from the corresponding author upon reasonable request.
